# Molecular Characterization of a Ryanodine Receptor Gene in the Rice Leaffolder, *Cnaphalocrocis medinalis* (Guenée)

**DOI:** 10.1371/journal.pone.0036623

**Published:** 2012-05-02

**Authors:** Jianjun Wang, Yanqing Li, Zhaojun Han, Youli Zhu, Zhijuan Xie, Jian Wang, Yaping Liu, Xianchun Li

**Affiliations:** 1 College of Horticulture and Plant Protection, Yangzhou University, Yangzhou, China; 2 Department of Entomology and BIO5 Institute, University of Arizona, Tucson, Arizona, United States of America; 3 College of Plant Protection, Nanjing Agricultural University, Nanjing, China; U. Kentucky, United States of America

## Abstract

Ryanodine receptors (RyRs) are the targets of two novel classes of synthetic insecticidal chemicals, phthalic acid diamides and anthranilic diamides. Isolation of full-length RyR cDNAs is a critical step towards the structural and functional characterization of insect RyRs and an understanding of the molecular mechanisms underlying the species selective toxicity of diamide insecticides. However, there has been little research on the insect RyR genes due to the high molecular weight of the RyR proteins. In this study, we isolated a full-length RyR cDNA (named as CmRyR) from *Cnaphalocrocis medinalis*, an important rice pest throughout Southeast Asia. The composite CmRyR gene contains an ORF of 15264 bp encoding a protein of 5087 amino acid residues, which shares 79% overall identity with its *Drosophila melanogaster* homologue. All hallmarks of the RyR proteins are conserved in the CmRyR protein, suggesting that CmRyR is a structural and functional analogue of known RyRs. A multiple sequence alignment illustrates that the insect RyRs share high levels of amino acid sequence identity at the the COOH-terminal region. However, the amino acid residues analogous to the CmRyR residues N^4922^, N^4924^, N^4935^, L^4950^, L^4981^, N^5013^ and T^5064^ are unique to lepidopteran RyRs compared with non-lepidopteran insect RyRs. This finding suggests that these residues may be involved in the differences in channel properties between lepidopteran and non-lepidopteran insect RyRs and in the species selective toxicity of diamide insecticides. Furthermore, two alternative splicing sites were identified in the CmRyR gene, one of which was located in the central part of the predicted second SPRY domain. Diagnostic PCR showed that the inclusion frequencies of two mutually exclusive exons (a/b) and one optional exon (c) differed between developmental stages or adult anatomical regions. Our results imply that alternative splicing may be a major means of generating functional diversity in *C. medinalis* RyR channel.

## Introduction

Developing insecticides that act on novel biochemical targets is important in crop protection due to the ability of insects to rapidly develop insecticide resistance. Calcium (Ca^2+^) is a key second messenger that regulates diverse cellular processes, including muscle contraction, hormone secretion, synaptic transmission, fertilization, and gene transcription. The process of muscle contraction involves the modulation of two distinct channel types: the voltage-gated channels, which regulate the entry of external calcium, the inositol 1,4,5-triphosphate receptor (IP_3_Rs) and the ryanodine receptor channels (RyRs), which regulate the release of calcium from intracellular stores. It has been suggested that insect calcium channels would offer an excellent insecticide target for commercial exploitation [1]–[2].

Recently, the development of two new classes of synthetic insecticidal chemicals with extremely high activity against a range of lepidopteran pest species has led to a renewed interest in this field. The first chemical class is comprised of phthalic acid diamides, including the compound flubendiamide which was co-developed by Nihon Nohyaku and Bayer CropScience [3]–[6]. The second chemical class is comprised of the anthranilic diamides, including the compound chlorantraniliprole (Rynaxypyr™) which was discovered and developed commercially by Dupont Crop Protection [7]–[9]. These diamides are potent activators of insect RyRs [10] and show exceptional selectivity for insect RyRs when compared to mammalian RyRs.

As the largest ion channels currently known, RyRs are located on the sarcoplasmic reticulum of muscles and the endoplasmic reticulum of neurons and many other cell types. To date, three types of ryanodine receptor (RyR1, RyR2 and RyR3) have been identified in mammals; each is the product of a separate gene [11]. RyR1 and RyR2 are predominately found in skeletal and cardiac muscles, respectively. RyR3 is ubiquitously expressed at low levels throughout different tissues but is relatively abundant in brain and certain skeletal tissues [12]–[14]. There is approximately 65% amino acid sequence identity between the mammalian RyR isoforms [15], and the sequence deviations mainly stem from three divergent regions of the RYR genes, referred to as DR1, DR2, and DR3 [16]. The RyRs are similar in structure with a large NH_2_-terminal domain and a small COOH-terminal domain protruding into the cytoplasm, as well as several transmembrane regions near the COOH-terminus. Due to alternative splicing, a variety of RyR variants have been isolated, and some show differences in their channel function properties [17]–[24].

Despite the important role of RyRs in the development of novel and selective insecticides, there have been very few studies on insect RyR genes. In 1994, Takeshima and colleagues described a 25.7 kb genomic DNA fragment that contained a gene for a *Drosophila melanogaster* RyR homologue (DmRyR) [25]. In all, 26 exons were found to encode the open reading frame and a predicted protein of 5216 or 5112 amino acids was reported. This receptor showed 45–47% identity with the three mammalian RyRs. Functional expression of *Drosophila* RyR has subsequently been reported in CHO cells [26] and a *Spodoptera frugiperda* cell line (Sf9) [7], [27], respectively. In addition to the *D. melanogaster* RyR, cDNAs encoding the COOH-terminal 1172 amino acids of a RyR gene (*Hv*-RyR) have been cloned and characterized from the lepidopteran pest *Heliothis virescens* [28]. Recently, cDNAs encoding two novel lepidopterous RyRs were cloned from the silkworm, *Bombyx mori* (sRyR), and the diamondback moth, *Plutella xylostella* (PxRyR), respectively [29]–[30]. In the present study, we isolated a full-length RyR cDNA (named CmRyR) from the rice leaffolder, *Cnaphalocrocis medinalis* (Lepidoptera: Pyralidae), an important rice insect pest throughout Southeast Asia. We report the structural features of CmRyR and document the developmentally regulated alternative splicing of CmRyR transcripts.

## Materials and Methods

### Insects

Adult rice leaffolders were collected in 2008 in the paddy field of the experimental farm at Yangzhou University and cultured in potted rice plants covered with an 80-mesh cage.

### Reverse transcriptase-polymerase chain reaction (RT-PCR)

**Figure 1:**
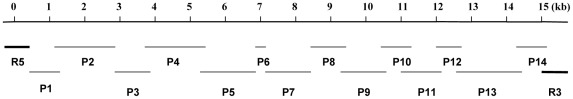
Schematic diagram of clones used to compile the complete CmRyR cDNA sequence. The top line indicates the full-length CmRyR cDNA in kilobases. Thin lines (P1–P14) represent CmRyR cDNA clones obtained by RT-PCR. Solid lines (R5 and R3) represent CmRyR cDNA clones obtained by RACE.

Total RNA from eggs (0–12 h old), first instar larvae (0–6 h old), third instar larvae, fifth instar larvae, pupae, and adult heads and bodies (0–24 h old), was extracted using an SV total RNA isolation system (Promega, Madison, WI), according to the manufacturer's instructions. First-strand cDNA was synthesized from 5 µg of total RNA using the Primescript™ First-Strand cDNA Synthesis kit (TaKaRa, Dalian, China) according to the manufacturer's instructions. Degenerate primer pairs were designed against the amino acid residues that were conserved between insects, other invertebrates, and vertebrate RyR isoforms (Table 1). PCR was performed in a 25 µl reaction volume containing 20–50 ng cDNA, 0.8 µM of each degenerate primer, 0.2 mM of each dNTP, 2 mM of MgCl_2_, 1.25 U Ex Taq™ polymerase (TaKaRa, Dalian, China) and 2.5 µL Ex Taq™ buffer (Takara, Dalian, China). A touchdown PCR protocol was used, which consisted of 1 cycle at 94°C for 5 min, 12 cycles at 94°C for 30 s, 52–41°C (decreasing by −1°C/cycle) for 30 s, and 72°C for 2 min, followed by 25 cycles of 94°C for 30 s, 40°C for 30 s, and 72°C for 2 min, and a final extension at 72°C for 10 min. Furthermore, specific primers were designed to amplify across the gaps (Table 1). A total of 14 RT-PCR products corresponding to the CmRyR cDNA sequence were finally obtained (Figure 1).

**Table 1:** Oligonucleotide primers used for RT-PCR, RACE and diagnostic PCR.

Primer name	Sequence (5′ to 3′)*	Description
258.RYRF13	TGGTGGACNGTNCAYCCNGC (WWTVHPA)	RT-PCR product P1
127.RYRR1	TCCATYTTNCCYTCYTCRTG (HEEGKMD)	
279.H2.2F2	TGGGCAAAGTGGAGGAGAAG	RT-PCR product P2
280.A1.1R1	CTCACTGTCTCCGAAGCCGT	
139.RYRF7	GARAAYACNCAYAAYYTNTGG (ENTHNLW)	RT-PCR product P3
141.RYRR7	TCCCARTTNGCYTTYTCCAT(MEKANWE)	
303.A1.1F1	GATACTCCACCTTGCCTG	RT-PCR product P4
143.RYRR8	TCRTTYTTRCANACYTCCAT(MEVCKNE)	
327.K3.9F1:	TGCGGCAAGGCTTCTATGAC	RT-PCR product P5
302.J1.6R1	CCATCAGACTGGAGTAAGCG	
290.RYRF14	GCNATGTTYGAYCAYTTYGA(AMFDHFD)	RT-PCR product P6
257.RYRR13	TCNCCRTTNACCCANACRCA(CVWVNGE)	
296.J1.6F1	GCAACTCGGAGCTGGTAG	RT-PCR product P7
239.A2.7R	CCCGTACACCCAACCATTCT	
146.RYRF10	GARCAYTAYCAYGAYGCNTGG(EHYHDAW)	RT-PCR product P8
147.RYRR10	GCNACCATYTCYTTYTCYTT(KEKEMVA)	
238.A2.7F1	CGCATCGCAACTACTTCATA	RT-PCR product P9
235.A3.7R1	CCACAACTGTAGTGACTCTGCG	
148.RYRF11	CCNTGGATGACNMGNATHGC(PWMTRIA)	RT-PCR product P10
149.RYRR11	TGYTGNGGRTGRTCDATCAT(MIDHPQQ)	
237.A3.7F2	GCCAAGATCATAGACGACACG	RT-PCR product P11
234.47.5R1	CCAAAGCCTGCTGGTTCTGT	
132.RYRF4	ATGGAYTTYTAYTGGCAYTA (MDFYWHY)	RT-PCR product P12
133.RYRR4	GGYTCRTTNGGCATRTGYTC(EHMPNEP)	
153.RYRSF1	GGACTACATCGGGTTCTGCG	RT-PCR product P13
155.RYRSR2	GGTCTTCTCCATGCCCAGCA	
134.RYRF5	GTNAAYTAYTGGGAYAARTT (VNYWDKF)	RT-PCR product P14
136.RYRR5	CATRTTCCANACRTANGTYTC(ETYVWNM)	
276.H2.2R1	TGGAAGAACCTCAGCACATCGC	5′ -RACE product R5
277.H2.2R2	ACACTAAGATCAAGTCGTCTCCCAC	
201.48F1	CAACAAGAACTGCCACGACA	3′ -RACE product R3
202.48F2	GAGCATAACTTGGCGAACTACA	
371.CmRyRF1	GTCAGTTCCGGGAAATGGTA	diagnostic PCR for exon a
372.CmRyRR1	CCCACTGCTTGCCAAACGAC	
373.CmRyRF2	CAGTTTCGGGCAGCAGTTCA	diagnostic PCR for exon b
374.CmRyRR2	ATCCTGGTATCTGGCATTTC	
375.CmRyRF3	AACAACGACCTCAACACCAT	diagnostic PCR for the absence of exon c
376.CmRyRR3	GTGGCTGAATCCGTACCACC	
377.CmRyRF4	TCGACCACCAGATGTCGTGA	diagnostic PCR for the presence of exon c
378.CmRyRR4	TATCGGTGAGGAGGTCGTAG	

* Corresponding amino acid sequences for degenerate primers were shown in parentheses.

### Rapid amplification of cDNA ends (RACE)

To complete the cDNA sequence of CmRyR, 5′ -RACE and 3′ -RACE reactions were performed using 5′-full RACE core set and 3′-full RACE core set (Takara, Dalian, China), respectively, following the manufacturer's instructions. Gene specific primers (GSP) used for the 5′ - and 3′ -RACE are listed in Table 1.

### Cloning and sequence analysis

RT-PCR and RACE products were cloned into the pMD18-T vector (TaKaRa, Dalian, China) and sequenced. Nucleotide sequences from individual clones were assembled into a full-length contig using the ContigExpress program, which is part of the Vector NTI Advance 9.1.0 (Invitrogen) suite of programs. Nucleotide residues were numbered, with the adenine of the first initiation codon being expressed as 1. The sequence alignment was performed using CLUSTALW [31] with the default settings. The aligned sequences were used to construct the phylogenetic tree in MEGA5 [32]. Transmembrane region predictions were made using the TMHMM Server v.2.0 (http://www.cbs.dtu.dk/services/TMHMM/). Conserved domains were predicted using the Conserved Domains Database (NCBI) or by alignment to other published RyRs.

### Diagnostic PCR analyses for the detection of alternative exons

Diagnostic PCR was used to detect the presence of each putative alternative exon in the individual cDNA clones. Table 1 lists the names and nucleotide sequences of the primers used in the diagnostic PCR reactions. Briefly, fragments containing the alternative exons were amplified by a set of primers flanking the alternative exon region. The PCR products were cloned into the pMD18-T vector (TaKaRa, Dalian, China), and a 2.5 µl aliquot of the overnight culture of each fragment clone was used as a template for diagnostic PCR. Mutually exclusive exons were identified using two exon-specific primers, one based on the sense strand sequence of one exon and the other on the antisense strand of the other exon. Optional exons were identified using a primer spanning the exon and flanking region or a primer spanning the flanking region excluding the exon sequence. These exon-specific primers were paired with counterpart primers located on either side of the alternatively spliced segment to generate unique PCR products representing the presence or absence of alternative exon in each clone. Amplicons were resolved on a 1% agarose gel, stained with ethidium bromide, and visualized with an UV transilluminator. Representative clones exhibiting unique banding patterns were sequenced to confirm the reliability of the diagnostic PCR assay.

### Database entries

The entire coding sequence of CmRyR has been deposited in the GenBank and the accession number is JQ799046.

## Results

### Cloning of CmRyR cDNA

**Figure 2:**
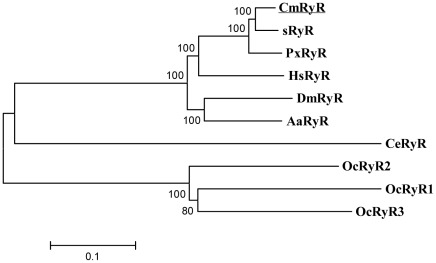
Phylogenetic tree of the RyR family. The CmRyR amino acid sequence was aligned to 9 representative RyR isoforms from 7 species and used for phylogenetic analysis. The Neighbor-joining tree was generated in MEGA5 with 1000 bootstrap replicates. RyR sequences are obtained from the following GenBank entries: DJ085056 for *Bombyx mori* (sRyR); AET09964 for *Plutella xylostella* (PxRyR); EFN78897 for *Harpegnathos saltator* (HsRyR); BAA41471 for *Drosophila melanogaster* (*DmRyR*); EAT44864 for *Aedes aegypti* (AaRyR); BAA08309 for *Caenorhabditis elegans* (CeRyR); CAA33279 for *Oryctolagus cuniculus* RyR1 (OcRyR1); NP_001076226 for *Oryctolagus cuniculus* RyR2 (OcRyR2); NP_001076231 for *Oryctolagus cuniculus* RyR3 (OcRyR3).

RT-PCR and RACE were used to amplify the entire coding sequence of the ryanodine receptor cDNA from *C. medinalis*. A total of 16 overlapping cDNA fragments were obtained (Figure 1). Compilation of the cDNA clones resulted in a 15,773-bp contiguous sequence containing a 15,264 bp ORF. The 289 bp of 5′-untranslated sequence upstream of the initiation codon is 41% GC and lacks a TATA box. The 220 bp of 3′-untranslated sequence downstream of the termination codon (TGA) is 65% AT but does not contain the canonical polyadenylation signal AAUAAA. The encoded 5,087 amino acid residues predict a protein with molecular mass of 574,339 Da, including the initiator methionine. An amino acid sequence alignment shows that CmRyR shares the greatest amino acid identity with sRyR (94%) and is 92% and 79% identical in pairwise comparisons with the PxRyR and DmRyR amino acid sequences, respectively. Identities of CmRyR with the three rabbit RyR isoforms are similar (45–46%). Phylogenetic analysis also reveals that CmRyR is grouped with sRyR and PxRyR, with high bootstrapping support in 1000 replications (Figure 2).

During the sequencing of these clones, a number of nucleotide differences were observed between overlapping clones. A total of 35 nucleotide substitution sites were found in the ORF, of which 20 nucleotide substitutions resulted in amino acid substitution, and 15 nucleotide substitutions were silent (Table 2). These polymorphisms were located both in the NH_2_- and COOH-terminal parts of CmRyR, and may represent different alleles or errors committed during the PCR procedure.

**Table 2:** Nucleotide polymorphisms of the CmRyR cDNA.

Nucleotide position*	Nucleotide exchange	Amino acid exchange#
486	A→G	Silent
837	C→T	Silent
1021	A→G	K^341^→E
3006	A→T	E^1002^→D
3042	A→G	Silent
3051	C→T	Silent
3060	C→G	Silent
3105	A→G	Silent
3582	T→C	Silent
6409	A→G	K^2137^→E
6424	A→G	T^2142^→A
6452	G→A	R^2151^→Q
6843	T→C	Silent
6868	T→G	C^2290^→G
6964	C→T	Silent
10252	A→G	T^3418^→A
10351	G→A	A^3451^→T
10521	C→T	Silent
10524	G→T	W^3508^→C
10554	A→G	Silent
10559	A→T	Y^3520^→F
10638	G→T	Silent
11076	G→C	Silent
11079	C→A	Silent
11127	C→G	Silent
11189	T→G	I^3730^→S
11705	T→C	V^3902^→A
11762	A→G	Q^3921^→R
11863	T→C	F^3955^→L
11915	T→C	V^3972^→A
11947	A→G	T^3983^→A
12724	G→A	G^4242^→S
14326	A→C	K^4776^→L
14327	A→T	K^4776^→L
15124	A→T	M^5042^→L

* The number of A in the initial methionine codon represents 1.

# Amino acids are written in one-letter code with their position in the amino acid sequence.

### Predicted domain structure of CmRyR

**Figure 3:**
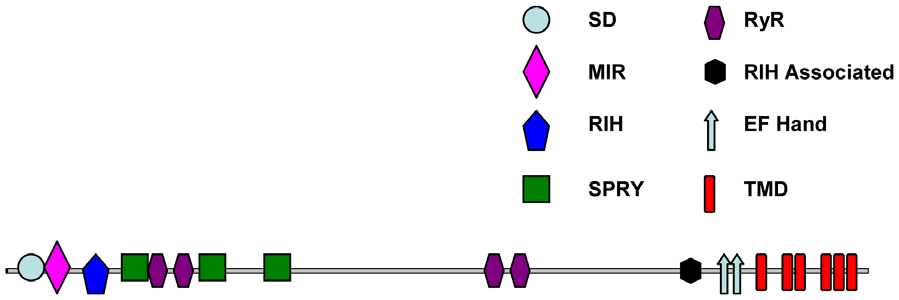
Analysis of CmRyR structure. Schematic showing the location of putative transmembrane domains (TMDs) and conserved structural domains identified using the Conserved Domains Database (NCBI), including: MIR (Mannosyltransferase, IP_3_R and RyR) domains (pfam02815), RIH (RyR and IP_3_R Homology) domains (pfam01365), SPRY (SPla and the RyR) domains (pfam00622), RyR domains (RyR repeated domain) (pfam02026), RIH-associated domains (RyR and IP_3_R Homology associated) (pfam08454), EF hands, and suppressor-domain-like domains (SD) (pfam08709).

The CmRyR amino acid sequence was analyzed for putative regulatory domains (Figure 3). The NH_2_-terminal region of CmRyR contains an MIR (Mannosyltransferase, IP_3_R and RyR) domain at positions 212–393, a RIH (RyR and IP_3_R Homology) domain at positions 440–649, and a suppressor-domain-like domain (SD) at positions 12–201, which showed high levels of homology to the suppressor domain of IP_3_Rs [33]. As with the mammalian RyRs, three copies of a SPRY (SPla and RyR) domain (666–803, 1092–1213, 1518–1659) and four copies of a RyR domain (854–948, 967–1061, 2802–2895, and 2928–3016) were also predicted. The RIH-associated domain preceding the C-terminal transmembrane regions was conserved and can be found at position 3975–4098. Six transmembrane helices (TM1 to TM6) were predicted at positions 4434–4456, 4615–4637, 4697–4719, 4839–4861, 4887–4909, and 4967–4986, respectively. Additionally, a consensus sequence for the ATP/GTP-binding motif (P-loop), [GA]XXXXGK[ST] [34], was found in the NH_2_-terminal region (1088–1095).

### Analysis of COOH-terminal region of CmRyR and comparison with known RyRs

**Figure 4:**
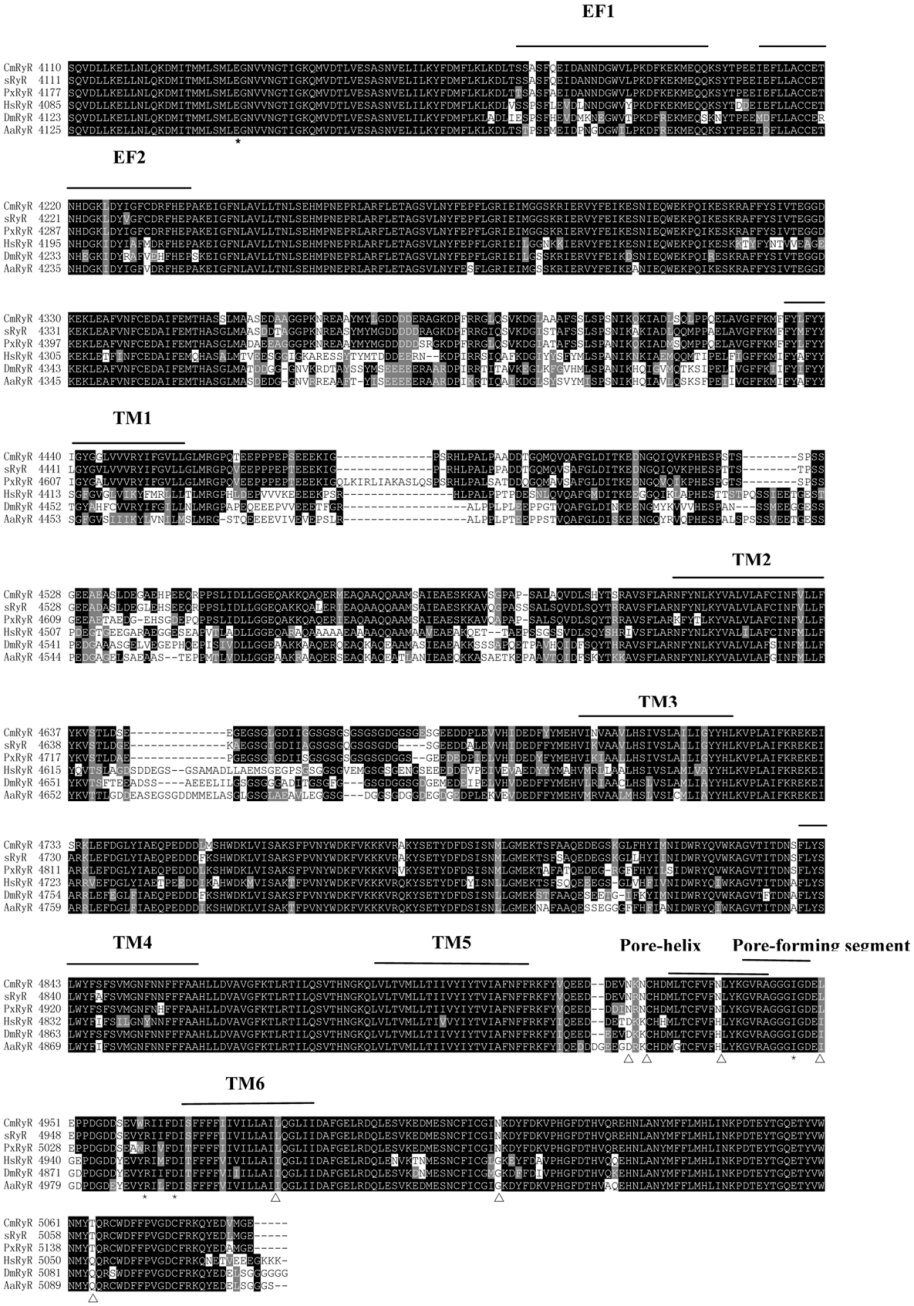
Alignment of COOH-terminal region of the insect RyR isoforms. Identical amino acids are shown in black boxes and similar amino acids are highlighted in gray boxes. Gaps have been introduced to permit alignment. Asterisks below the alignment indicate the position of residues discussed in the text (E^4134^, I^4946^, R^4962^, and D^4966^ of CmRyR). Triangles below the alignment indicate unique residues (N^4922^, N^4924^, N^4935^, L^4950^, L^4981^, N^5013^ and T^5064^ of CmRyR) in the lepidopteran homologues. Abbreviations and GenBank entries for the RyR isoforms are described in Figure 2.

The COOH-terminal region of RyR has been shown to be functionally important [35], and the insecticide flubendiamide is mainly incorporated into the transmembrane domains (amino acids 4111–5084) of the *B. mori* sRyR [29]. Therefore, the COOH-terminal region of CmRyR was compared with other reported insect RyRs, including sRyR from *B. mori*, PxRyR from *P. xylostella*, HsRyR from *Harpegnathos saltator*, DmRyR from *D. melanogaster*, and AaRyR from *Aedes aegypti* (Figure 4). The alignment showed that the six transmembrane regions in CmRyR exhibited high sequence identity to other insect RyRs. The pore helix, which is analogous to the P loop of the voltage-activated Ca^2+^, Na^+^ and K^+^ channels [36], was predicted to be in the loop region between the putative fifth and sixth transmembrane helices of the receptors. The sequence motif, GXRXGGGXGD, which constitutes part of the pore-forming segments of the Ca^2+^ release channels [37], was also highly conserved in CmRyR (4939–4948) and other insect RyRs. Two consensus Ca^2+^-binding EF-hand motifs originally reported in the lobster RyR [38] were also present in tandem at positions 4175–4202 and 4210–4237. A glutamate residue proposed to be involved in the Ca^2+^ sensitivity in rabbit RyR3 (E^3885^) [39] and RyR1 (E^4032^) [40] was also detected in CmRyR (E^4134^) and other insect RyRs. Additionally, residues corresponding to I^4897^, R^4913^, and D^4917^ of rabbit RyR1, which were recently shown to play an important role in the activity and conductance of the Ca^2+^release channel [41], were conserved in CmRyR (I^4946^, R^4962^, D^4966^) and other insect RyR isoforms.

Despite the significant similarity, CmRyR and other lepidopteran homologues show some potentially important sequence divergence from the non-lepidopteran insects in the COOH-terminal region, especially in close proximity to the pore-forming segment. For example, the lepidopteran homologues differ from the non-lepidopteran RyRs at positions analogous to CmRyR residues N^4922^, N^4924^, N^4935^, L^4950^, L^4981^, N^5013^ and T^5064^ (Figure 4). To verify this finding, a total of 31 RyR isoform sequences from 26 species were aligned, including 5 lepidopteran homologues, 13 non-lepidopteran insect homologues, 5 other invertebrate homologues, 2 non-mammalian vertebrate homologues, and 6 mammalian homologues. The alignment showed that seven CmRyR residues (N^4922^, N^4924^, N^4935^, L^4950^, L^4981^, N^5013^ and T^5064^) were highly conserved in the lepidopteran homologues, while different residues at the corresponding positions (D^4942^, K^4944^, H^4955^, I^4970^, I^5001^, G^5033^ and Q^5084^, respectively, in DmRyR) were shared by all the 13 non-lepidopteran insect RyRs (Figure S1). Surprisingly, residues at these positions are highly conserved in the non-lepidopteran insect, other invertebrate, and vertebrate RyRs, except that one residue in *Tetranychus urticae* (N^5026^) and one residue in *Caenorhabditis elegans* (L^4933^) were identical to lepidopteran homologues (N^4935^ and L^4950^ in CmRyR), respectively (Figure S1). Our results suggest that these residues might be involved in the differences in channel properties between lepidopteran and non-lepidopteran insect RyRs and in the species selective toxicity of diamide insecticides.

### Developmental regulation of alternative exon usage

**Figure 5:**
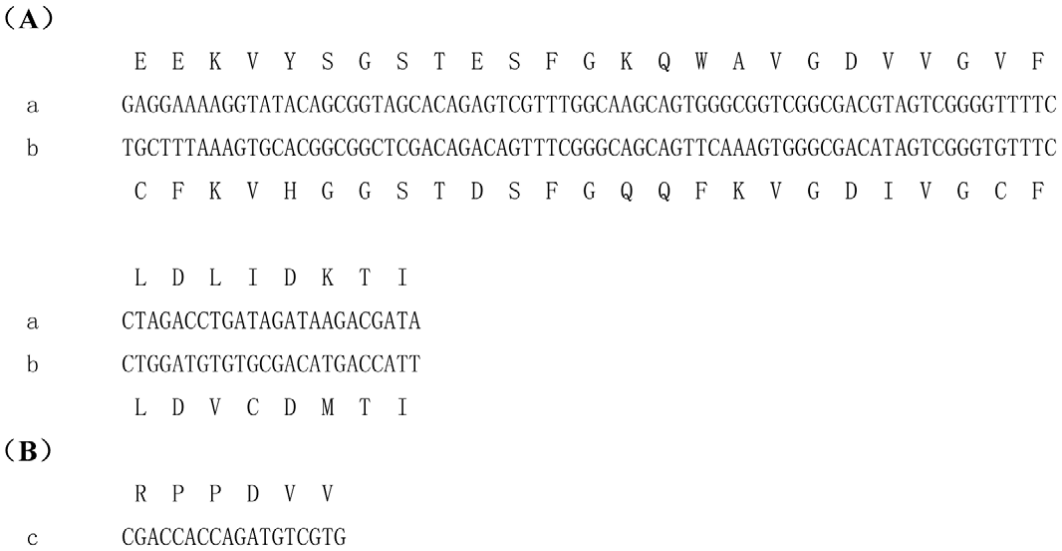
Nucleotide and inferred amino acid sequences of alternative exons in the CmRyR gene.

The alignment of multiple cDNA clone sequences revealed two putative alternative splice sites in CmRyR, named insect alternative splicing I (IASI) and insect alternative splicing II (IASII). IASI is located between amino acid residues 1136–1168 and forms one pair of mutually exclusive exons (a/b), which was also reported in *Drosophila* [25]. IASII is located between amino acid residues 2915–2920 and forms the optional exon c. Figure 5 shows the nucleotide and inferred amino acid sequences of the three alternative exons identified in the present study. Interestingly, IASI corresponds to the central part of the predicted second SPRY domain, while IASII is located between the predicted third and fourth RyR domains.

**Figure 6:**
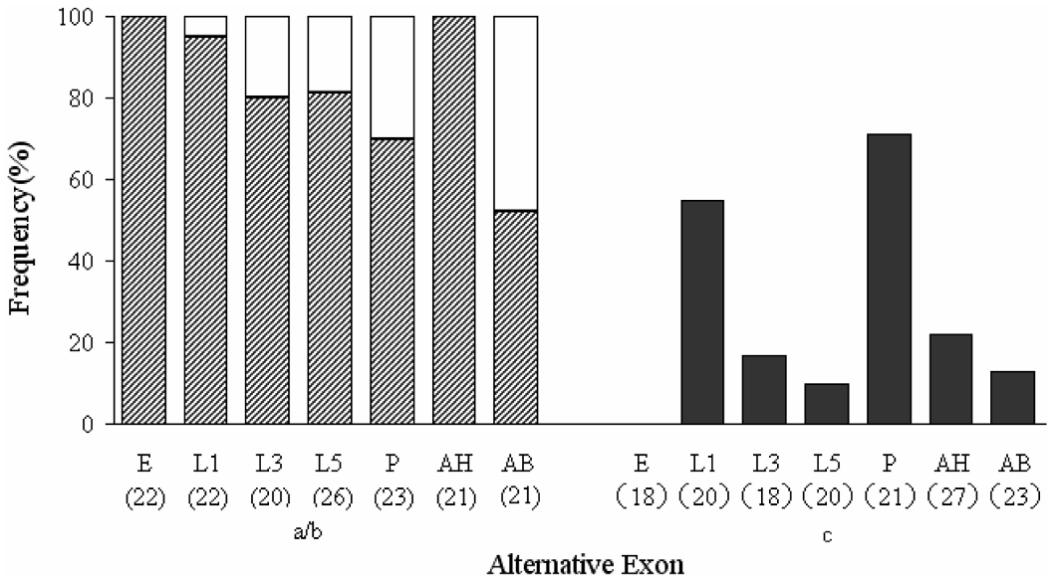
Relative frequencies of individual CmRyR alternative exon usage in eggs (E), first instar larvae (L1), third instar larvae (L3), fifth instar larvae (L5), pupae (P), adult heads (AH), and adult bodies (AB). Mutually exclusive exons are represented by hatched bars (exon a) and open bars (exon b). Optional exon c is indicated by black bars. Numbers in parentheses represent the total numbers of clones investigated.

Diagnostic PCR was used to determine the usage of each putative alternative exon in seven discrete mRNA pools: eggs, first instar larvae (0–6 h old), third instar larvae, fifth instar larvae, pupae, 0–24 h adult heads, and 0–24 h adult bodies. Data were collected from sets of 18–27 clones for each fragment and developmental stage. The usage frequencies of each putative alternative exon for the seven mRNA pools are summarized in Figure 6. The results show that the usage of the mutually exclusive exons (a/b) exhibited marked developmental and anatomical regulation. Exon a was present in all cDNA clones examined from the egg and adult head cDNA pools, but only in 16 of the 23 clones and 11 of the 21 clones analyzed from the pupal and adult body cDNA pools, respectively. In contrast, exon b was not detected in the egg and adult head cDNA pools, but was present at low to moderate frequencies (30% and 48%) in the pupal and adult body cDNA pools. Developmental regulation of exon inclusion was also observed for exon c. It was present at low frequencies (0%, 17%, and 10%) in eggs, third instar larvae and fifth instar larvae, respectively, but at moderate to high frequencies (55% and 71%) in first instar larvae and pupae.

## Discussion

RyR is known to release Ca^2+^ from the sarcoplasmic reticulum to induce muscle contraction in various vertebrate and invertebrate muscles. The structural divergence between the mammalian and the insect RyR isoforms implies that RyRs may serve as potential targets for potent insecticides with low mammalian toxicity. Recently, two new classes of synthetic insecticidal chemicals, phthalic acid diamides and anthranilic diamides, have been developed. Treatment with both phthalic and anthranilic diamides were reported to elicit intracellular Ca^2+^ release in isolated insect neurons [3]–[4], [7]. These RyR modulators exhibit exceptional insecticidal activity against lepidopteran insect pests and show a high selectivity for insect RyRs compared to mammalian RyRs. However, the basis of this selectivity is not yet understood.

Isolating the full-length RyR cDNA is a critical step towards a comprehensive structural and functional characterization of insect RyRs and could lead to an understanding of the molecular mechanisms that underlie the species selective toxicity of diamide insecticides. However, this approach has been hampered by the high molecular weight of the RyR isoforms. To the best of our knowledge, full-length RyR homologues have been cloned and characterized in only three insect species: *D. melanogaster*, *B. mori*, and *P. xylostella*. In the present study, we cloned and sequenced the full-length cDNA encoding the RyR from *C. medinalis*. The deduced amino acid sequence of CmRyR shows a reasonable degree of identity with known RyRs throughout the entire molecule and also shares several common structural features. Two consensus Ca^2+^-binding EF-hands are present in the COOH-terminus of the CmRyR at positions 4183–4194 and 4285–4296, suggesting that this channel, similar to mammalian RyRs, may be regulated by cytosolic Ca^2+^. The close similarity of the pore regions of CmRyR and mammalian RyRs, including the conserved GGGXGD selectivity filter motif, suggests that CmRyR likely forms functional cation channels with a high single-channel conductance and permeability to Ca^2+^. Molecular phylogenetic analyses also confirm that the cloned cDNA encodes a RyR isoform that is most closely related to the sRyR isoform (Figure 2). Taken together, these results suggest that CmRyR is a structural and functional analogue of other known RyRs.

While the exact binding sites for flubendiamide or chlorantraniliprole have not yet been identified, binding studies on the microsomal membranes from insect muscles suggest that flubendiamide and chlorantraniliprole act at a site distinct from the ryanodine binding site localized in the pore of the insect RyR complex [4], [7]. Recently, it was found that flubendiamide mainly incorporates into the transmembrane domain (amino acids 4111–5084) of sRyR [29]. In this study, alignment of the deduced amino acid sequences of the COOH-terminal region of six insect RyRs illustrates that insect RyRs share high levels of sequence identity, especially surrounding the TM5 and TM6 regions. Sequence analysis, however, uncovered amino acid residues with marked uniqueness in this region in lepidopteran insects. For example, amino acid residues at positions analogous to the CmRyR residues N^4922^, N^4924^, N^4935^, L^4950^, L^4981^, N^5013^ and T^5064^ are unique to lepidopteran RyR homologues, whereas the corresponding residues in non-lepidopteran insect RyRs are highly conserved with other invertebrate and vertebrate RyRs. Therefore, it is thought that these residues might be involved in the differences in channel properties between lepidopteran and non-lepidopteran insect RyRs and in the species selective toxicity of diamide insecticides. Further studies, including mutagenesis and a heterologous expression of various mutagenized RyRs, are needed to address this interesting hypothesis.

Alternative splicing is a key posttranscriptional processing mechanism that generates structural and functional diversity, leading to the specialization of many membrane proteins, including sodium and calcium channels. A total of nine alternative splice sites (a, b, c/d, i, j, e, f, h, l/k) have been identified in the *para* sodium channel in *D. melanogaster* [42]. In mammalians, more than 10 distinct splice variants have been identified in RyR isoforms from human, rabbit, mouse, mink, and dog. Some splice variants of RyR isoforms were found to predominantly suppress Ca^2+^ release or to contribute to distinct Ca^2+^ releasing patterns [22]–[24], [43]–[45]. For example, one smooth muscle-specific human RyR3 splice variant, which lacks a 29 amino acid fragment (His^4406^–Lys^4434^) encompassing a predicted transmembrane helix, acts as a tissue-specific dominant negative regulator of RyR2 channels via the formation of heteromeric channel complexes [22]. Similarly, one human RyR2 splice variant, which inclues a 24 bp exon, protected cells from caffeine-evoked apoptosis through its negative effects on intracellular Ca^2+^ release [24]. Recent studies with peptides corresponding to the RyR1 splice variants ASI (+) or ASI (−) suggest that the ASI region (Ala^3481^-Gln^3485^) in the RyR1 channel contributes to an inhibitory module in RyR1 that influences EC coupling [44].

In contrast to the three RyR genes in mammals, only one gene (Rya-r44F) encoding a ryanodine receptor is present in *D. melanogaster*, and it is likely that the major means of generating diversity in insect RyR channels involves alternative splicing. In fact, alternative splicing of the RyR transcript has been reported in *D. melanogaster* [25]. In this study, two putative alternative splice sites were identified in the CmRyR gene. One of the two alternative splice sites, IASI, is conserved in *D. melanogaster* [25], and the other alternative splice site, IASII, is also found in *Helicoverpa armigera* (Wang et al., unpublished data). The expression profiles of the CmRyR splice variants exhibit marked developmental or anatomical regulation. Interestingly, IASI was located in the central part of the predicted second SPRY domain (SPRY2). The SPRY domain structure was first described in the *Dictyostelium discoideum* tyrosine kinase spore lysis A (SplA) and the mammalian RyRs [46]. SPRY domains are widely regarded to be protein-protein interaction domains that are involved in a wide spectrum of biological functions, including the regulation of cytokine signaling and innate retroviral restriction [47]. Recently, the SPRY2 domain in RyR1 has been identified as an in vitro binding partner for the II–III loop of the *α*_1S_ subunit of the skeletal muscle dihydropryidine receptor (DHPR) [48]. Because the sequences of the mutually exclusive exons a and b in CmRyR were highly divergent, differing at 13 of 33 amino acid residues (Figure 5), it is quite likely that the CmRyR splicing variants generated by IASI may have different protein-protein interactions. In summary, while the properties and functions of the CmRyR splice variants remain to be studied, our results imply that alternative splicing may play an important role in the spatial and temporal coding of Ca^2+^ signals in insects, and this signaling may greatly influence cellular function and phenotype.

## Supporting Information

Figure S1 **Alignment of amino acid sequence of partial COOH-terminal region in 31 RyR isoforms from 26 species.** Identical amino acids are shown in black boxes and similar amino acids are highlighted in gray boxes. Gaps have been introduced to permit alignment. Triangles below the alignment indicate unique residues (N^4922^, N^4924^, N^4935^, L^4950^, L^4981^, N^5013^ and T^5064^ of CmRyR) for lepdopteran homologues. Abbreviations and GenBank entries for sRyR, PxRyR, HsRyR, DmRyR, AaRyR, CeRyR, OcRyR1, OcRyR2, OcRyR3 isoforms are described in Fig. 2. The other RyR sequences are obtained from the following GenBank entries: AAD01425 for *Heliothis virescens* (HvRyR); EHJ77857 for *Danaus plexippus* (DpRyR); EEB11809 for *Pediculus humanus corporis* (PhcRyR); EEZ99829 for *Tribolium castaneum* (TcRyR); AF483192 for *Periplaneta americana* (PaRyR); EAA13701 for *Anopheles gambiae* (AgRyR); XP_392217 for *Apis mellifera* (AmRyR); XP_003484552 for *Bombus impatiens* (BiRyR); XP_003393894 for *Bombus terrestris* (BtRyR); EFN67324 for *Camponotus floridanus* (CfRyR); XP_001842971 for *Culex quinquefasciatus* (CqRyR); XP_003246190 for *Acyrthosiphon pisum* (ApRyR); BAK26392 for *Tetranychus urticae* (TuRyR);XP_002578860 for *Schistosoma mansoni* (SmRyR); CAX69439 for *Schistosoma japonicum* (SjRyR); BAB84714 for *Hemicentrotus pulcherrimus* (HpRyR); BAA04646 for *Rana catesbeiana* RyRα (RcRyRα); BAA04647 for *Rana catesbeiana* RyRβ (RcRyRβ); P21817 for human RyR1 (hRyR1); Q92736 for human RyR2 (hRyR2); Q15413 for human RyR3 (hRyR3).
(DOC)
