# Supplementary material for: Molecular Characterization of a Ryanodine Receptor Gene in the Rice Leaffolder, Cnaphalocrocis medinalis (Guenée)
Source: PLoS One. 2012 May 2;7(5):e36623. doi: 10.1371/journal.pone.0036623 (PMC3342285; doi:10.1371/journal.pone.0036623)
Supplement: Figure S1 — Alignment of amino acid sequence of partial COOH-terminal region in 31 RyR isoforms from 26 species. Identical amino acids are shown in black boxes and similar amino acids are highlighted in gray boxes. Gaps have been introduced to permit alignment. Triangles below the alignment indicate unique residues (N4922, N4924, N4935, L4950, L4981, N5013 and T5064 of CmRyR) for lepdopteran homologues. Abbreviations and GenBank entries for sRyR, PxRyR, HsRyR, DmRyR, AaRyR, CeRyR, OcRyR1, OcRyR2, OcRyR3 isoforms are described in Fig. 2. The other RyR sequences are obtained from the following GenBank entries: AAD01425 for Heliothis virescens (HvRyR); EHJ77857 for Danaus plexippus (DpRyR); EEB11809 for Pediculus humanus corporis (PhcRyR); EEZ99829 for Tribolium castaneum (TcRyR); AF483192 for Periplaneta americana (PaRyR); EAA13701 for Anopheles gambiae (AgRyR); XP_392217 for Apis mellifera (AmRyR); XP_003484552 for Bombus impatiens (BiRyR); XP_003393894 for Bombus terrestris (BtRyR); EFN67324 for Camponotus floridanus (CfRyR); XP_001842971 for Culex quinquefasciatus (CqRyR); XP_003246190 for Acyrthosiphon pisum (ApRyR); BAK26392 for Tetranychus urticae (TuRyR);XP_002578860 for Schistosoma mansoni (SmRyR); CAX69439 for Schistosoma japonicum (SjRyR); BAB84714 for Hemicentrotus pulcherrimus (HpRyR); BAA04646 for Rana catesbeiana RyRα (RcRyRα); BAA04647 for Rana catesbeiana RyRβ (RcRyRβ); P21817 for human RyR1 (hRyR1); Q92736 for human RyR2 (hRyR2); Q15413 for human RyR3 (hRyR3). (DOC) [file pone.0036623.s001.doc]

CmRyR 4887 LVLTVMLLTIIVYIYTVIAFNFFRKFYVQEEDD---EVNKNCHDMLTCFVFNLYKGVRAGGGIGDELEPPDGDDSEVWRIIFDISFFFFIIVILLAILQGLIIDAFGELR
sRyR 4884 LVLTVMLLTIIVYIYTVIAFNFFRKFYVQEEDD---EVNRNCHDMLTCFVFNLYKGVRAGGGIGDELEPPDGDDSEVYRIIFDISFFFFIIVILLAILQGLIIDAFGELR
PxRyR 4964 LVLTVMLLTIIVYIYTVIAFNFFRKFYVQEEDD---DINRNCHDMLTCFVFNLYKGVRAGGGIGDELEPPDGDDSEAWRIVFDITFFFFIIVILLAILQGLIIDAFGELR
HvRyR 326 LVLTVMLLTIIVYIYTVIAFNFFRKFYVQEEDD---EVNRNCHDMLTCFVFNLYKGVRAGGGIGDELEPPDGDDSEVYRIIFDISFFFFIIVILLAILQGLIIDAFGELR
DpRyR 1 -----MLLTIIVYIYTVIAFNFFRKFYVQEEDD---EVNRNCHDMLTCFVFNLYKGVRAGGGIGDELEPPDGDDSEVYRIIFDISFFFFVIVILLAILQGLIIDAFGELR
HsRyR 4876 LVLTVMLLTIVVYIYTVIAFNFFRKFYIQEEDD---ETDKKCHHMLTCFVFHLYKGVRAGGGIGDEIGEPDGDDYEVYRIMFDITFFFFVIVILLAIIQGLIIDAFGELR
DmRyR 4907 LVLTVMLLTIIVYIYTVIAFNFFRKFYIQEEDE---EVDKKCHDMLTCFVFHLYKGVRAGGGIGDEIGDPDGDDYEVYRIIFDITFFFFVIIILLAIIQGLIIDAFGELR
AaRyR 4913 LVLTVMLLTIIVYIYTVIAFNFFRKFYIQEDDDG-EEGDRKCHDMGTCFVFHLYKGVRAGGGIGDEIGDPDGDEYEVYRILFDISFFFFVIVILLAIIQGLIIDAFGELR
PhcRyR 4855 LILTVMLLTIIVYIYTVIAFNFFRKFYVQEEDD---EVDKKCHNMLTCFVFHLYKGVRAGGGIGDEIEPPDGDEYEVYRILFDITFFFFVIVILLAIIQGLIIDAFGELR
TcRyR 4723 LVLTVMLLTIVVYIYTVIAFNFFRKFYVQEEDE---EVDKKCHDMLTCFVFHLYKGVRAGGGIGDEIEPPDGDDYEVYRIMFDITFFFFVIVILLAIIQGLIIDAFGELR
PaRyR 112 LVLTVMLLTIIVYIYTVIAFNFFRKFYVQEED---EEVDKKCHDMLTCFVFHLYKGVRAGGGIGDEIEPPDGDDYEVYRILFDITFFFFVIVILLAIIQGLIIDAFGELR
AgRyR 4904 LVLTVMLLTIIVYIYTVIAFNFFRKFYVQEDDDG-EEGDRKCHDMATCFVFHLYKGVRAGGGIGDEIGDPDGDEYEVYRILFDITFFFFVIVILLAIIQGLIIDAFGELR
AmRyR 4878 LVLTVMLLTIVVYIYTVIAFNFFRKFYIQEEDD---EVDKKCHDMLTCFVFHLYKGVRAGGGIGDEIGEPDGDDYEVYRIMFDITFFFFVIVILLAIIQGLIIDAFGELR
BiRyR 4904 LVLTVMLLTIVVYIYTVIAFNFFRKFYIQEEDD---EVDKKCHDMLTCFVFHLYKGVRAGGGIGDEIGEPDGDDYEVYRIMFDITFFFFVIVILLAIIQGLIIDAFGELR
BtRyR 4904 LVLTVMLLTIVVYIYTVIAFNFFRKFYIQEEDD---EVDKKCHDMLTCFVFHLYKGVRAGGGIGDEIGEPDGDDYEVYRIMFDITFFFFVIVILLAIIQGLIIDAFGELR
CfRyR 2928 LVLTVMLLTIVVYIYTVIAFNFFRKFYIQEEDD---ETDKKCHHMLTCFVFHLYKGVRAGGGIGDEIGEPDGDDYEVYRIMFDITFFFFVIVILLAIIQGLIIDAFGELR
CqRyR 1133 LVLTVMLLTIIVYIYTVIAFNFFRKFYIQEDDDG-EEGDRKCHDMGTCFVFHLYKGVRAGGGIGDEIGDPDGDEYEVYRILFDISFFFFVIVILLAIIQGLIIDAFGELR
ApRyR 4897 LVLTVLLLTIIVYIYTVIAFNFFRKFYVQEEDD---EVDKKCHDMLTCFVFHLYKGVRAGGGIGDEIGSPDGDDYEVYRIMFDITFFFFVIIILLAIIQGLIIDAFGELR
TuRyR 4975 LVLTVMLLIIVVYMYTVIAFNFFRKFYVQGDDDEENEPDKKCHSMLTCFVFNIYQGVRAGGGIGDVIEPPDGDEYEVYRIVFDITFFVFVIIILVAIIQGLIIDAFGELR
SmRyR 4799 --QAIMLTSIVIYLYTVVAFNFFRKFYVK-DNDG--VPDPKCNDMKTCFIFHLHTGLRAGGGIGDEIEAPDGDESENYRILFDLTFFFFVIIILLAIIQGLIIDAFGDLR
SjRyR 370 LVLTVMLTSIVIYLYTVVAFNFFRKFYVK-DNDG--VPDPKCNDMKTCFIFHLHTGLRAGGGIGDEIEAPDGDESESYRILFDLTFFFFVIIILLAIIQGLIIDAFGDLR
CeRyR 4868 LILTIMMTLVVVYLYTVIAFNFFRKFYVQEGEEG-EEPDRKCHNMLTCFIYHFYAGVRAGGGIGDELESPYGDDLEYPRMFYDISFFFFVIIILLAIMQGLIIDAFGELR
HpRyR 5118 LVLTLMMTCVIIYLYTVLAFNFFRKFYTK-DDEG--EIEYKCHNMMSCFVFHLHSGLRAGGGIADEIEAPDGDVYEYYRIIFDITFFFFVIVILLAIIQGLIIDAFGELR
RcRyRα 4837 LMMTVGLLAVVVYLYTVVAFNFFRKFYNKSEDED--EPDMKCDDMMTCYLFHMYVGVRAGGGIGDEIEDPAGDEYELYRVVFDITFFFFVIVILLAIIQGLIIDAFGELR
RcRyRβ 4668 LVLTVGLLAVVVYLYTVVAFNFFRKFYNKSEDED--DPDMKCDDMMTCYLFHMYVGVRAGGGIGDEIEDPAGDPYEMYRIVFDITFFFFVIVILLAIIQGLIIDAFGELR
OcRyR1 4837 LVMTVGLLAVVVYLYTVVAFNFFRKFYNKSEDED--EPDMKCDDMMTCYLFHMYVGVRAGGGIGDEIEDPAGDEYELYRVVFDITFFFFVIVILLAIIQGLIIDAFGELR
OcRyR2 4768 LVLTVGLLAVVVYLYTVVAFNFFRKFYNKSEDGD--TPDMKCDDMLTCYMFHMYVGVRAGGGIGDEIEDPAGDEYEIYRIIFDITFFFFVIVILLAIIQGLIIDAFGELR
OcRyR3 4672 LVLTVGLLAVVVYLYTVVAFNFFRKFYNKSEDDD--EPDMKCDDMMTCYLFHMYVGVRAGGGIGDEIEDPAGDPYEMYRIVFDITFFFFVIVILLAIIQGLIIDAFGELR
hRyR1 4838 LVMTVGLLAVVVYLYTVVAFNFFRKFYNKSEDED--EPDMKCDDMMTCYLFHMYVGVRAGGGIGDEIEDPAGDEYELYRVVFDITFFFFVIVILLAIIQGLIIDAFGELR
hRyR2 4767 LVLTVGLLAVVVYLYTVVAFNFFRKFYNKSEDGD--TPDMKCDDMLTCYMFHMYVGVRAGGGIGDEIEDPAGDEYEIYRIIFDITFFFFVIVILLAIIQGLIIDAFGELR
hRyR3 4670 LVLTVGLLAVVVYLYTVVAFNFFRKFYNKSEDDD--EPDMKCDDMMTCYLFHMYVGVRAGGGIGDEIEDPAGDPYEMYRIVFDITFFFFVIVILLAIIQGLIIDAFGELR
 △ △ △ △ △

CmRyR 4994 DQLESVKEDMESNCFICGINKDYFDKVPHGFDTHVQREHNLANYMFFLMHLINKPDTEYTGQETYVWNMYTQRCWDFFPVGDCFRKQYEDVMGE-----
sRyR 4991 DQLESVKEDMESNCFICGINKDYFDKVPHGFDTHVQREHNLANYMFFLMHLINKPDTEYTGQETYVWNMYTQRCWDFFPVGDCFRKQYEDLMGE-----
PxRyR 5071 DQLESVKEDMESNCFICGINKDYFDKVPHGFDTHVQREHNLANYMFFLMHLINKPDTEYTGQETYVWNMYTQRCWDFFPVGDCFRKQYEDAMGE-----
HvRyR 433 DQLESVKEDMESNCFICGINKDYFDKVPHGFDTHVQREHNLANYMFFLMHLINKPDTEYTGQETYVWNMYTQRCWDFFPVGDCFRKQYEDLMGE-----
DpRyR 103 DQLESVKEDMESNCFICGINKDYFDKVPHGFDTHVQREHNLANYMFFLMHLINKPDTEYTGQETFVWNMYTQRCWDFFPVGDCFRKQYEDAMGE-----
HsRyR 4983 DQLENVKTNMESNCFICGLGKEYFDAVPHGFDTHVQQEHNLANYMFFLMHLINKPDTEYTGQETYVWNMYQQRCWDFFPVGDCFRKQNETVEEEGKKK-
DmRyR 5014 DQLESVKDNMESNCFICGMGKDFFDIVPHGFDTHVQKEHNLANYMFFLMHLINKPDTEYTGQETYVWNMYQQRSWDFFPVGDCFRKQYEDELSGGGGGG
AaRyR 5022 DQLESVKEDMESNCFICGIGKDYFDKVPHGFDTHVAQEHNLANYMFFLMHLINKPDTEYTGQETYVWNMYQQRCWDFFPVGDCFRKQYEDELSGGGS--
PhcRyR 4962 DQLESVKEDMESNCFICGIGKDYFDKVPHGFDTHVAQEHNLANYMFFLMHLINKPDTEYTGQETYVWNMYQQRCWDFFPVGDCFRKQYEDELGGGNN--
TcRyR 4830 DQLESVKEDMESNCFICGMGKEYFDKVPHGFDTHVQQEHNLANYMFFLMHLINKPDTEYTGQETYVWNMYQQRCWDFFPVGDCFRKQYEEELGGGGG--
PaRyR 219 DQLESVKDDMESNCFICGIGKDYFDKVPHGFDTHVAQEHNLANYMFFLMHLINKPDTEYTGQETYVWNMYQQRCWDFFP--------------------
AgRyR 5013 DQLESVKEDMESNCFICGIGKDYFDKVPHGFDTHVAQEHNLANYMFFLMHLINKPDTEYTGQETYVWNMYQQRCWDFFPVGDCFRKQYEDELGGGGS--
AmRyR 4985 DQLENVKTNMESNCFICGLGKEYFDTVPHGFDTHVQQEHNLANYMFFLMHLINKPDTEYTGQETYVWNMYQQRCWDFFPVGDCFRKQNEAVEEEAKKK-
BiRyR 5011 DQLENVKTNMESNCFICGLGKEYFDTVPHGFDTHVQQEHNLANYMFFLMHLINKPDTEYTGQETYVWNMYQQRCWDFFPVGDCFRKQNEAVEEEAKKK-
BtRyR 5011 DQLENVKTNMESNCFICGLGKEYFDTVPHGFDTHVQQEHNLANYMFFLMHLINKPDTEYTGQETYVWNMYQQRCWDFFPVGDCFRKQNEAVEEEAKKK-
CfRyR 3035 DQLDNVKTNMESNCFICGLGKEYFDAVPHGFDTHVQQEHNLANYMFFLMHLINKPDTEYTGQETYVWNMYQQRCWDFFPVGDCFRKQNETVEEEAKKK-
CqRyR 1242 DQLESVKEDMESNCFICGIGKDYFDKVPHGFDTHVAQEHNLANYMFFLMHLINKPDTEYTGQETYVWNMYQQRCWDFFPVGDCFRKQYEDELGGGGSS-
ApRyR 5004 DQLESVKEDMESNCFICGIGKDYFDKVPHGFDTHVQEEHNLANYMFFLMHLINKPDTEYTGQETYVWNMYQQRCWDFFPVGDCFRKQYEEELGGGGGV-
TuRyR 5085 GQLQSVVDDMEANCFICGIGKDYFDKTPHGFETHVMKEHNLANYLFFLMHLINKPDTDYTGQETYVWELYQKRCWDFFPVGECFRKQYEDELSGAS---
SmRyR 4904 DQLEQVKEDLESKCFICGIGKEYFDKIPHGFEQHVEKEHNFANYMYFLMHIINKPDTEYTGQETYVWELYQQRCWDFFPIGDCFRKQYEEELQPK----
SjRyR 477 DQLEQVKEDLESKCFICGIGKEYFDKIPHGFEQHVEKEHNFANYMYFLMHIINKPDTEYTGQETYVWELYQQRCWDFFPIGDCFRKQYEEELQPK----
CeRyR 4977 DQQESATEKLESSCFICDIGKETFDRMPRGFEIHTTKEHNFANYLFFLQHLVNKDETEYTGQETYVREKYDNRDWDFFPVGECFVKQYEDQLLQS----
HpRyR 5225 DQLEQVREDMETKCFICSIGREYFDKLPHGFELHTSKEHDLSNYMFFLMYLINKPETEHTGQESYVWQLYQQRCWDFFPVGDCFRKQYDDEQA------
RcRyRα 4945 DQQEQVKEDMETKCFICGIGSDYFDTTPHGFETHTLEEHNLANYMFFLMYLINKDDTEHTGQESYVWKMYQERCWDFFPAGDCFRKTYEDQLG------
RcRyRβ 4776 DQQEQVREDMETKCFICGIGNDYFDTTPHGFETHTLQEHNLANYLFFLMYIINKDETEHTGQESYVWKMYQERCWDFFPAGDCFRKQYEDQLG------
OcRyR1 4945 DQQEQVKEDMETKCFICGIGSDYFDTTPHGFETHTLEEHNLANYMFFLMYLINKDETEHTGQESYVWKMYQERCWDFFPAGDCFRKQYEDQLS------
OcRyR2 4876 DQQEQVKEDMETKCFICGIGNDYFDTVPHGFETHTLQEHNLANYLFFLMYLINKDETEHTGQESYVWKMYQERCWEFFPAGDCFRKQYEDQLN------
OcRyR3 4780 DQQEQVREDMETKCFICGIGNDYFDTTPHGFETHTLQEHNLANYLFFLMYLINKDETEHTGQESYVWKMYQERCWDFFPAGDCFRKQYEDQLG------
hRyR1 4946 DQQEQVKEDMETKCFICGIGSDYFDTTPHGFETHTLEEHNLANYMFFLMYLINKDETEHTGQESYVWKMYQERCWDFFPAGDCFRKQYEDQLS------
hRyR2 4875 DQQEQVKEDMETKCFICGIGNDYFDTVPHGFETHTLQEHNLANYLFFLMYLINKDETEHTGQESYVWKMYQERCWEFFPAGDCFRKQYEDQLN------
hRyR3 4778 DQQEQVREDMETKCFICGIGNDYFDTTPHGFETHTLQEHNLANYLFFLMYLINKDETEHTGQESYVWKMYQERCWDFFPAGDCFRKQYEDQLG------
 △ △

**Figure S1. Alignment of amino acid sequence of partial COOH-terminal region in 31 RyR isoforms from 26 species.** Identical amino acids are shown in black boxes and similar amino acids are highlighted in gray boxes. Gaps have been introduced to permit alignment. Triangles below the alignment indicate unique residues (N^4922^, N^4924^, N^4935^, L^4950^, L^4981^, N^5013^ and T^5064^ of CmRyR) for lepdopteran homologues. Abbreviations and GenBank entries for sRyR, PxRyR, HsRyR, DmRyR, AaRyR, CeRyR, OcRyR1, OcRyR2, OcRyR3 isoforms are described in Fig. 2. The other RyR sequences are obtained from the following GenBank entries: AAD01425 for *Heliothis virescens* (HvRyR); EHJ77857 for *Danaus plexippus* (DpRyR)；EEB11809 for *Pediculus humanus corporis* (PhcRyR); EEZ99829 for *Tribolium castaneum* (TcRyR); AF483192 for *Periplaneta americana* (PaRyR); EAA13701 for *Anopheles gambiae* (AgRyR); XP_392217 for *Apis mellifera* (AmRyR); XP_003484552 for *Bombus impatiens* (BiRyR); XP_003393894 for *Bombus terrestris* (BtRyR); EFN67324 for *Camponotus floridanus* (CfRyR); XP_001842971 for *Culex quinquefasciatus* (CqRyR); XP_003246190 for *Acyrthosiphon pisum* (ApRyR); BAK26392 for *Tetranychus urticae* (TuRyR)；XP_002578860 for *Schistosoma mansoni* (SmRyR); CAX69439 for *Schistosoma japonicum* (SjRyR); BAB84714 for *Hemicentrotus pulcherrimus* (HpRyR); BAA04646 for *Rana catesbeiana* RyRα (RcRyRα); BAA04647 for *Rana catesbeiana* RyRβ (RcRyRβ); P21817 for human RyR1 (hRyR1); Q92736 for human RyR2 (hRyR2); Q15413 for human RyR3 (hRyR3).
